# Supplementary material for: Multi‐dataset identification of innovative feature genes and molecular mechanisms in keratoconus
Source: J Cell Mol Med. 2024 Sep 19;28(18):e70079. doi: 10.1111/jcmm.70079 (PMC11412914; doi:10.1111/jcmm.70079)
Supplement: Supplementary file 2 — Material S2. [file JCMM-28-e70079-s002.zip › 7.GSEA analysis/6.SNORA74B/KEGG_NEUROACTIVE_LIGAND_RECEPTOR_INTERACTION.html]

Details for gene set KEGG\_NEUROACTIVE\_LIGAND\_RECEPTOR\_INTERACTION[GSEA]

|  || Dataset | SNORA74B.group.cls#h\_versus\_l |
| Phenotype | group.cls#h\_versus\_l |
| Upregulated in class | h |
| GeneSet | KEGG\_NEUROACTIVE\_LIGAND\_RECEPTOR\_INTERACTION |
| Enrichment Score (ES) | 0.21753868 |
| Normalized Enrichment Score (NES) | 0.7889275 |
| Nominal p-value | 0.9979424 |
| FDR q-value | 1.0 |
| FWER p-Value | 1.0 |
Table: GSEA Results Summary

  

Fig 1: Enrichment plot: KEGG\_NEUROACTIVE\_LIGAND\_RECEPTOR\_INTERACTION      
 Profile of the Running ES Score & Positions of GeneSet Members on the Rank Ordered List

  

| SYMBOL | TITLE | RANK IN GENE LIST | RANK METRIC SCORE | RUNNING ES | CORE ENRICHMENT || 1 | GRID1 | na | 123 | 0.859 | 0.0125 | Yes |
| 2 | TRPV1 | na | 130 | 0.851 | 0.0283 | Yes |
| 3 | TSHR | na | 171 | 0.799 | 0.0422 | Yes |
| 4 | TRHR | na | 222 | 0.746 | 0.0547 | Yes |
| 5 | GNRHR | na | 570 | 0.579 | 0.0555 | Yes |
| 6 | NPY2R | na | 649 | 0.556 | 0.0637 | Yes |
| 7 | CHRNB2 | na | 755 | 0.532 | 0.0706 | Yes |
| 8 | DRD5 | na | 761 | 0.530 | 0.0804 | Yes |
| 9 | CHRNA4 | na | 1041 | 0.480 | 0.0813 | Yes |
| 10 | GABRG3 | na | 1079 | 0.475 | 0.0892 | Yes |
| 11 | GRIK2 | na | 1124 | 0.469 | 0.0967 | Yes |
| 12 | HTR6 | na | 1157 | 0.463 | 0.1045 | Yes |
| 13 | LEP | na | 1168 | 0.461 | 0.1129 | Yes |
| 14 | DRD3 | na | 1191 | 0.457 | 0.1208 | Yes |
| 15 | ADRA1D | na | 1329 | 0.435 | 0.1250 | Yes |
| 16 | MLNR | na | 1373 | 0.429 | 0.1318 | Yes |
| 17 | GABRR2 | na | 1454 | 0.420 | 0.1374 | Yes |
| 18 | GLP1R | na | 1478 | 0.417 | 0.1445 | Yes |
| 19 | MC1R | na | 1641 | 0.403 | 0.1474 | Yes |
| 20 | GALR2 | na | 1744 | 0.394 | 0.1518 | Yes |
| 21 | HCRTR2 | na | 1847 | 0.385 | 0.1561 | Yes |
| 22 | MTNR1A | na | 2033 | 0.368 | 0.1576 | Yes |
| 23 | CNR1 | na | 2170 | 0.357 | 0.1604 | Yes |
| 24 | GABRA3 | na | 2232 | 0.353 | 0.1652 | Yes |
| 25 | HTR2C | na | 2383 | 0.341 | 0.1673 | Yes |
| 26 | ADORA1 | na | 2464 | 0.335 | 0.1712 | Yes |
| 27 | HTR4 | na | 2500 | 0.332 | 0.1764 | Yes |
| 28 | BDKRB2 | na | 2580 | 0.327 | 0.1803 | Yes |
| 29 | THRB | na | 2640 | 0.324 | 0.1846 | Yes |
| 30 | CHRNA2 | na | 2743 | 0.317 | 0.1876 | Yes |
| 31 | NPY4R | na | 2772 | 0.315 | 0.1927 | Yes |
| 32 | GABRA2 | na | 2933 | 0.305 | 0.1938 | Yes |
| 33 | CHRNA1 | na | 2981 | 0.302 | 0.1981 | Yes |
| 34 | OPRM1 | na | 3073 | 0.297 | 0.2011 | Yes |
| 35 | HTR1A | na | 3386 | 0.282 | 0.1973 | Yes |
| 36 | GRM5 | na | 3432 | 0.281 | 0.2013 | Yes |
| 37 | GRM4 | na | 3462 | 0.279 | 0.2057 | Yes |
| 38 | GABRG1 | na | 3522 | 0.277 | 0.2092 | Yes |
| 39 | CGA | na | 3650 | 0.272 | 0.2106 | Yes |
| 40 | TACR3 | na | 4447 | 0.245 | 0.1921 | Yes |
| 41 | RXFP1 | na | 4536 | 0.241 | 0.1940 | Yes |
| 42 | RXFP2 | na | 4539 | 0.241 | 0.1985 | Yes |
| 43 | OPRK1 | na | 4594 | 0.239 | 0.2014 | Yes |
| 44 | GRIK1 | na | 4768 | 0.233 | 0.2008 | Yes |
| 45 | ADRA2B | na | 4803 | 0.232 | 0.2041 | Yes |
| 46 | P2RX6 | na | 4910 | 0.228 | 0.2053 | Yes |
| 47 | SSTR1 | na | 4957 | 0.227 | 0.2083 | Yes |
| 48 | ADORA3 | na | 5043 | 0.224 | 0.2100 | Yes |
| 49 | PTGER3 | na | 5065 | 0.223 | 0.2136 | Yes |
| 50 | P2RY10 | na | 5129 | 0.221 | 0.2159 | Yes |
| 51 | SCTR | na | 5215 | 0.218 | 0.2175 | Yes |
| 52 | C5AR1 | na | 5480 | 0.209 | 0.2138 | No |
| 53 | CHRNA10 | na | 5872 | 0.196 | 0.2061 | No |
| 54 | GH1 | na | 5883 | 0.196 | 0.2095 | No |
| 55 | SSTR4 | na | 6170 | 0.187 | 0.2047 | No |
| 56 | P2RY6 | na | 6284 | 0.183 | 0.2049 | No |
| 57 | P2RY2 | na | 6442 | 0.178 | 0.2037 | No |
| 58 | P2RY13 | na | 6453 | 0.178 | 0.2068 | No |
| 59 | AGTR2 | na | 6504 | 0.177 | 0.2086 | No |
| 60 | HTR5A | na | 6596 | 0.173 | 0.2092 | No |
| 61 | S1PR3 | na | 6662 | 0.172 | 0.2106 | No |
| 62 | GRIA2 | na | 6702 | 0.171 | 0.2126 | No |
| 63 | GALR1 | na | 6917 | 0.165 | 0.2095 | No |
| 64 | GABBR1 | na | 6919 | 0.165 | 0.2126 | No |
| 65 | LPAR3 | na | 6971 | 0.163 | 0.2142 | No |
| 66 | LTB4R | na | 7050 | 0.161 | 0.2149 | No |
| 67 | SSTR3 | na | 7229 | 0.156 | 0.2127 | No |
| 68 | CHRNB4 | na | 7253 | 0.155 | 0.2149 | No |
| 69 | F2RL3 | na | 7457 | 0.150 | 0.2118 | No |
| 70 | NPFFR2 | na | 7891 | 0.139 | 0.2019 | No |
| 71 | GHR | na | 7932 | 0.138 | 0.2033 | No |
| 72 | ADRA2C | na | 7948 | 0.138 | 0.2054 | No |
| 73 | CNR2 | na | 8013 | 0.136 | 0.2061 | No |
| 74 | NTSR1 | na | 8163 | 0.132 | 0.2043 | No |
| 75 | CHRM5 | na | 8349 | 0.128 | 0.2013 | No |
| 76 | MC5R | na | 8623 | 0.122 | 0.1957 | No |
| 77 | GRIN2A | na | 8634 | 0.122 | 0.1977 | No |
| 78 | GPR156 | na | 8666 | 0.121 | 0.1990 | No |
| 79 | GLRA1 | na | 8729 | 0.120 | 0.1995 | No |
| 80 | CCKAR | na | 8822 | 0.118 | 0.1990 | No |
| 81 | GRIN3A | na | 9023 | 0.113 | 0.1953 | No |
| 82 | LPAR1 | na | 9070 | 0.112 | 0.1961 | No |
| 83 | F2RL1 | na | 9590 | 0.101 | 0.1829 | No |
| 84 | PRSS3 | na | 9671 | 0.099 | 0.1825 | No |
| 85 | GRIN2D | na | 9698 | 0.099 | 0.1836 | No |
| 86 | HCRTR1 | na | 9703 | 0.099 | 0.1853 | No |
| 87 | HRH3 | na | 10021 | 0.092 | 0.1778 | No |
| 88 | GIPR | na | 10062 | 0.091 | 0.1784 | No |
| 89 | TAAR6 | na | 10351 | 0.086 | 0.1717 | No |
| 90 | LPAR6 | na | 10359 | 0.086 | 0.1731 | No |
| 91 | GABRA1 | na | 10500 | 0.083 | 0.1706 | No |
| 92 | NTSR2 | na | 11009 | 0.075 | 0.1572 | No |
| 93 | S1PR5 | na | 11066 | 0.074 | 0.1570 | No |
| 94 | GCGR | na | 11218 | 0.071 | 0.1539 | No |
| 95 | CHRNE | na | 11236 | 0.071 | 0.1548 | No |
| 96 | GLRB | na | 11543 | 0.066 | 0.1471 | No |
| 97 | GRM6 | na | 11560 | 0.066 | 0.1479 | No |
| 98 | C3AR1 | na | 11581 | 0.066 | 0.1486 | No |
| 99 | EDNRB | na | 11740 | 0.064 | 0.1452 | No |
| 100 | GABRB2 | na | 11790 | 0.063 | 0.1449 | No |
| 101 | PTGIR | na | 11810 | 0.063 | 0.1456 | No |
| 102 | S1PR1 | na | 11950 | 0.061 | 0.1427 | No |
| 103 | CYSLTR2 | na | 11979 | 0.060 | 0.1430 | No |
| 104 | GH2 | na | 12078 | 0.059 | 0.1412 | No |
| 105 | CSH1 | na | 12125 | 0.058 | 0.1410 | No |
| 106 | PTAFR | na | 12255 | 0.057 | 0.1383 | No |
| 107 | F2R | na | 12299 | 0.056 | 0.1381 | No |
| 108 | GABRB3 | na | 12337 | 0.056 | 0.1381 | No |
| 109 | TSPO | na | 12431 | 0.054 | 0.1364 | No |
| 110 | GABRA6 | na | 12538 | 0.053 | 0.1343 | No |
| 111 | TAAR2 | na | 12721 | 0.050 | 0.1300 | No |
| 112 | TAAR5 | na | 12938 | 0.048 | 0.1246 | No |
| 113 | ADRB2 | na | 13437 | 0.042 | 0.1109 | No |
| 114 | HTR2B | na | 13781 | 0.038 | 0.1017 | No |
| 115 | GABRR1 | na | 13961 | 0.036 | 0.0972 | No |
| 116 | LTB4R2 | na | 14097 | 0.035 | 0.0939 | No |
| 117 | LPAR4 | na | 14156 | 0.034 | 0.0929 | No |
| 118 | TAAR1 | na | 14240 | 0.033 | 0.0911 | No |
| 119 | DRD4 | na | 14278 | 0.033 | 0.0906 | No |
| 120 | GPR50 | na | 14398 | 0.031 | 0.0878 | No |
| 121 | TAAR9 | na | 14719 | 0.027 | 0.0790 | No |
| 122 | CHRM2 | na | 15078 | 0.024 | 0.0691 | No |
| 123 | PARD3 | na | 15192 | 0.023 | 0.0662 | No |
| 124 | MAS1 | na | 15197 | 0.023 | 0.0665 | No |
| 125 | PTH2R | na | 15445 | 0.020 | 0.0597 | No |
| 126 | BRS3 | na | 15567 | 0.019 | 0.0566 | No |
| 127 | PRLHR | na | 15842 | 0.016 | 0.0489 | No |
| 128 | HTR1F | na | 15877 | 0.016 | 0.0482 | No |
| 129 | OPRD1 | na | 16121 | 0.013 | 0.0414 | No |
| 130 | LHB | na | 16543 | 0.009 | 0.0294 | No |
| 131 | GZMA | na | 16674 | 0.008 | 0.0258 | No |
| 132 | PRL | na | 16840 | 0.006 | 0.0211 | No |
| 133 | TAAR8 | na | 16842 | 0.006 | 0.0212 | No |
| 134 | NPY1R | na | 16984 | 0.005 | 0.0172 | No |
| 135 | GABRG2 | na | 17032 | 0.004 | 0.0159 | No |
| 136 | HTR1E | na | 17068 | 0.004 | 0.0149 | No |
| 137 | GABRA4 | na | 18026 | -0.006 | -0.0127 | No |
| 138 | PLG | na | 18128 | -0.007 | -0.0155 | No |
| 139 | CHRNB1 | na | 18348 | -0.010 | -0.0217 | No |
| 140 | TACR2 | na | 18463 | -0.011 | -0.0248 | No |
| 141 | GRM7 | na | 18547 | -0.012 | -0.0269 | No |
| 142 | FPR2 | na | 18730 | -0.014 | -0.0320 | No |
| 143 | ADRA2A | na | 18870 | -0.016 | -0.0357 | No |
| 144 | TSHB | na | 18919 | -0.016 | -0.0368 | No |
| 145 | ADRB1 | na | 19094 | -0.018 | -0.0415 | No |
| 146 | GABRA5 | na | 19129 | -0.019 | -0.0421 | No |
| 147 | GRID2 | na | 19687 | -0.025 | -0.0578 | No |
| 148 | GHSR | na | 19707 | -0.025 | -0.0579 | No |
| 149 | CHRNA9 | na | 19806 | -0.026 | -0.0603 | No |
| 150 | TACR1 | na | 20017 | -0.029 | -0.0658 | No |
| 151 | GRIN2C | na | 20279 | -0.031 | -0.0728 | No |
| 152 | F2 | na | 20714 | -0.037 | -0.0847 | No |
| 153 | TBXA2R | na | 20979 | -0.040 | -0.0916 | No |
| 154 | NMUR2 | na | 21121 | -0.041 | -0.0949 | No |
| 155 | HTR1B | na | 21508 | -0.046 | -0.1053 | No |
| 156 | CHRND | na | 21675 | -0.048 | -0.1092 | No |
| 157 | DRD1 | na | 21750 | -0.049 | -0.1104 | No |
| 158 | NR3C1 | na | 21833 | -0.050 | -0.1119 | No |
| 159 | P2RX2 | na | 22038 | -0.052 | -0.1168 | No |
| 160 | AVPR1B | na | 22332 | -0.056 | -0.1243 | No |
| 161 | OPRL1 | na | 22375 | -0.056 | -0.1244 | No |
| 162 | P2RY14 | na | 22635 | -0.060 | -0.1308 | No |
| 163 | P2RX3 | na | 22657 | -0.060 | -0.1303 | No |
| 164 | CTSG | na | 22930 | -0.064 | -0.1370 | No |
| 165 | GALR3 | na | 23055 | -0.066 | -0.1393 | No |
| 166 | GRIN3B | na | 23321 | -0.070 | -0.1457 | No |
| 167 | FSHB | na | 23701 | -0.076 | -0.1553 | No |
| 168 | AVPR2 | na | 23814 | -0.078 | -0.1571 | No |
| 169 | GRPR | na | 23860 | -0.079 | -0.1569 | No |
| 170 | FPR3 | na | 23990 | -0.081 | -0.1591 | No |
| 171 | GRIN2B | na | 24135 | -0.083 | -0.1618 | No |
| 172 | NPFFR1 | na | 24155 | -0.083 | -0.1607 | No |
| 173 | ADORA2A | na | 24281 | -0.085 | -0.1628 | No |
| 174 | HRH1 | na | 24846 | -0.096 | -0.1773 | No |
| 175 | GRIK4 | na | 24916 | -0.097 | -0.1775 | No |
| 176 | CHRNA3 | na | 24935 | -0.097 | -0.1762 | No |
| 177 | ADRA1A | na | 25082 | -0.100 | -0.1786 | No |
| 178 | NMBR | na | 25087 | -0.100 | -0.1768 | No |
| 179 | GRIN1 | na | 25109 | -0.100 | -0.1755 | No |
| 180 | OXTR | na | 25294 | -0.103 | -0.1789 | No |
| 181 | P2RX4 | na | 25303 | -0.104 | -0.1772 | No |
| 182 | UTS2R | na | 25382 | -0.105 | -0.1775 | No |
| 183 | CALCRL | na | 25422 | -0.106 | -0.1767 | No |
| 184 | HTR7 | na | 25743 | -0.112 | -0.1839 | No |
| 185 | GRM1 | na | 25756 | -0.112 | -0.1821 | No |
| 186 | P2RX5 | na | 25838 | -0.113 | -0.1823 | No |
| 187 | CCKBR | na | 25960 | -0.116 | -0.1837 | No |
| 188 | HRH2 | na | 26044 | -0.118 | -0.1839 | No |
| 189 | S1PR4 | na | 26054 | -0.118 | -0.1819 | No |
| 190 | GLP2R | na | 26085 | -0.118 | -0.1806 | No |
| 191 | BDKRB1 | na | 26112 | -0.119 | -0.1791 | No |
| 192 | HTR1D | na | 26187 | -0.121 | -0.1790 | No |
| 193 | PTGER2 | na | 26203 | -0.121 | -0.1771 | No |
| 194 | PTH1R | na | 26609 | -0.130 | -0.1864 | No |
| 195 | ADORA2B | na | 26690 | -0.132 | -0.1863 | No |
| 196 | SSTR2 | na | 26716 | -0.133 | -0.1845 | No |
| 197 | P2RY1 | na | 26720 | -0.133 | -0.1821 | No |
| 198 | S1PR2 | na | 26731 | -0.133 | -0.1799 | No |
| 199 | P2RY4 | na | 26761 | -0.134 | -0.1782 | No |
| 200 | CHRM3 | na | 26903 | -0.138 | -0.1797 | No |
| 201 | GRM8 | na | 27332 | -0.148 | -0.1894 | No |
| 202 | GRIK5 | na | 27467 | -0.150 | -0.1904 | No |
| 203 | GABRE | na | 27574 | -0.153 | -0.1906 | No |
| 204 | THRA | na | 27581 | -0.154 | -0.1879 | No |
| 205 | LPAR2 | na | 27668 | -0.156 | -0.1875 | No |
| 206 | CRHR1 | na | 27732 | -0.158 | -0.1864 | No |
| 207 | ADCYAP1R1 | na | 27890 | -0.162 | -0.1879 | No |
| 208 | GABBR2 | na | 27897 | -0.162 | -0.1850 | No |
| 209 | GPR35 | na | 28002 | -0.165 | -0.1849 | No |
| 210 | VIPR2 | na | 28050 | -0.166 | -0.1832 | No |
| 211 | GHRHR | na | 28121 | -0.168 | -0.1821 | No |
| 212 | HTR2A | na | 28242 | -0.172 | -0.1823 | No |
| 213 | NPBWR2 | na | 28245 | -0.172 | -0.1792 | No |
| 214 | GRM2 | na | 28302 | -0.173 | -0.1775 | No |
| 215 | MC3R | na | 28559 | -0.181 | -0.1816 | No |
| 216 | MC4R | na | 28738 | -0.187 | -0.1832 | No |
| 217 | LEPR | na | 28912 | -0.192 | -0.1846 | No |
| 218 | CHRNG | na | 29345 | -0.205 | -0.1933 | No |
| 219 | PTGFR | na | 29444 | -0.208 | -0.1923 | No |
| 220 | NPY5R | na | 29473 | -0.209 | -0.1892 | No |
| 221 | AVPR1A | na | 29627 | -0.213 | -0.1896 | No |
| 222 | CHRNA6 | na | 29628 | -0.213 | -0.1856 | No |
| 223 | GRIA3 | na | 29711 | -0.216 | -0.1839 | No |
| 224 | CHRNA5 | na | 29713 | -0.216 | -0.1799 | No |
| 225 | AGTR1 | na | 29794 | -0.218 | -0.1781 | No |
| 226 | CALCR | na | 30132 | -0.231 | -0.1836 | No |
| 227 | CHRM1 | na | 30177 | -0.232 | -0.1805 | No |
| 228 | GRIA1 | na | 30738 | -0.255 | -0.1920 | No |
| 229 | CHRNA7 | na | 30927 | -0.264 | -0.1925 | No |
| 230 | PTGER1 | na | 31136 | -0.275 | -0.1933 | No |
| 231 | APLNR | na | 31188 | -0.277 | -0.1896 | No |
| 232 | HRH4 | na | 31198 | -0.278 | -0.1847 | No |
| 233 | GLRA3 | na | 31341 | -0.285 | -0.1834 | No |
| 234 | VIPR1 | na | 31506 | -0.295 | -0.1826 | No |
| 235 | NPBWR1 | na | 31521 | -0.296 | -0.1775 | No |
| 236 | DRD2 | na | 31542 | -0.297 | -0.1725 | No |
| 237 | GABRB1 | na | 31575 | -0.299 | -0.1678 | No |
| 238 | LHCGR | na | 31585 | -0.300 | -0.1624 | No |
| 239 | GRM3 | na | 31642 | -0.303 | -0.1584 | No |
| 240 | P2RX1 | na | 31891 | -0.317 | -0.1596 | No |
| 241 | GABRQ | na | 32246 | -0.337 | -0.1636 | No |
| 242 | CHRM4 | na | 32284 | -0.339 | -0.1583 | No |
| 243 | SSTR5 | na | 32308 | -0.341 | -0.1525 | No |
| 244 | KISS1R | na | 32382 | -0.346 | -0.1482 | No |
| 245 | CYSLTR1 | na | 32414 | -0.349 | -0.1425 | No |
| 246 | MCHR1 | na | 32426 | -0.349 | -0.1363 | No |
| 247 | P2RY8 | na | 32458 | -0.351 | -0.1306 | No |
| 248 | MTNR1B | na | 32579 | -0.359 | -0.1273 | No |
| 249 | GABRP | na | 32589 | -0.360 | -0.1208 | No |
| 250 | PRLR | na | 32623 | -0.362 | -0.1150 | No |
| 251 | FSHR | na | 32734 | -0.371 | -0.1112 | No |
| 252 | ADRA1B | na | 32758 | -0.373 | -0.1049 | No |
| 253 | MC2R | na | 32908 | -0.386 | -0.1020 | No |
| 254 | CHRNB3 | na | 32998 | -0.393 | -0.0972 | No |
| 255 | GPR83 | na | 33000 | -0.394 | -0.0898 | No |
| 256 | GRIK3 | na | 33021 | -0.395 | -0.0830 | No |
| 257 | F2RL2 | na | 33025 | -0.396 | -0.0756 | No |
| 258 | EDNRA | na | 33192 | -0.413 | -0.0727 | No |
| 259 | GABRD | na | 33358 | -0.431 | -0.0694 | No |
| 260 | ADRB3 | na | 33378 | -0.434 | -0.0618 | No |
| 261 | P2RX7 | na | 33412 | -0.440 | -0.0545 | No |
| 262 | GLRA2 | na | 33493 | -0.449 | -0.0484 | No |
| 263 | PRSS1 | na | 33618 | -0.467 | -0.0432 | No |
| 264 | PTGDR | na | 33947 | -0.523 | -0.0429 | No |
| 265 | FPR1 | na | 34045 | -0.544 | -0.0355 | No |
| 266 | GRIA4 | na | 34068 | -0.548 | -0.0259 | No |
| 267 | MCHR2 | na | 34091 | -0.554 | -0.0161 | No |
| 268 | CRHR2 | na | 34106 | -0.558 | -0.0061 | No |
| 269 | PTGER4 | na | 34266 | -0.599 | 0.0006 | No |
| 270 | NMUR1 | na | 34467 | -0.683 | 0.0076 | No |
Table: GSEA details [plain text format]

  

Fig 2: KEGG\_NEUROACTIVE\_LIGAND\_RECEPTOR\_INTERACTION      
 Blue-Pink O' Gram in the Space of the Analyzed GeneSet

  

Fig 3: KEGG\_NEUROACTIVE\_LIGAND\_RECEPTOR\_INTERACTION: Random ES distribution      
 Gene set null distribution of ES for **KEGG\_NEUROACTIVE\_LIGAND\_RECEPTOR\_INTERACTION**
